# The Multifaceted Role of Endothelial Sirt1 in Vascular Aging: An Update

**DOI:** 10.3390/cells13171469

**Published:** 2024-09-01

**Authors:** Roberto Campagna, Laura Mazzanti, Veronica Pompei, Sonila Alia, Arianna Vignini, Monica Emanuelli

**Affiliations:** 1 Department of Clinical Sciences, Polytechnic University of Marche, 60100 Ancona, Italy; v.pompei@staff.univpm.it (V.P.); s.alia@pm.univpm.it (S.A.); a.vignini@univpm.it (A.V.); m.emanuelli@univpm.it (M.E.); 2 Fondazione Salesi, Ospedale G. Salesi, 60100 Ancona, Italy; 3 Research Center of Health Education and Health Promotion, Università Politecnica delle Marche, 60100 Ancona, Italy

**Keywords:** Sirtuin 1, Sirt1, vascular aging, endothelial senescence

## Abstract

NAD^+^-dependent deacetylase sirtuin-1 (Sirt1) belongs to the sirtuins family, known to be longevity regulators, and exerts a key role in the prevention of vascular aging. By aging, the expression levels of Sirt1 decline with a severe impact on vascular function, such as the rise of endothelial dysfunction, which in turn promotes the development of cardiovascular diseases. In this context, the impact of Sirt1 activity in preventing endothelial senescence is particularly important. Given the key role of Sirt1 in counteracting endothelial senescence, great efforts have been made to deepen the knowledge about the intricate cross-talks and interactions of Sirt1 with other molecules, in order to set up possible strategies to boost Sirt1 activity to prevent or treat vascular aging. The aim of this review is to provide a proper background on the regulation and function of Sirt1 in the vascular endothelium and to discuss the recent advances regarding the therapeutic strategies of targeting Sirt1 to counteract vascular aging.

## 1. Introduction

The term sirtuins stands for Silent Information Regulator-SIRT proteins and includes proteins which are highly conserved class III NAD^+^-dependent histone deacetylases (HDACs). HDACs deacetylate lysine residues use nicotinamide adenine dinucleotide (NAD^+^) as a co-enzyme [1]. Traditionally, their deacetylation activity was thought to be directed to histone proteins, although it has been recently demonstrated that sirtuins exert a wide range of enzymatic activity, including deacetylation of non-histone proteins, demalonylation, desuccinylation, demyristoylation, and mono-adenosine diphosphate (ADP)-ribosylation [2]. The mammalian Sirt1 family contains seven enzymes (Sirt1–7) grouped into four classes: class I includes Sirt1–3, class II includes Sirt4, class III includes Sirt5, and class IV includes Sirt6–7 [3]. The intracellular localization of sirtuins is strictly related to their biological function. Indeed, while Sirt1, Sirt6, and Sirt7 are localized in the nucleus, Sirt3, Sirt4, and Sirt5 are located in the mitochondria, and Sirt2 is mainly expressed in the cytoplasm [4]. Due to their deacetylation activity occurring at the post-translational level, sirtuins may modulate a number of cellular pathways, including mitochondrial bioenergetic metabolism, cell cycle progression, homeostasis, DNA repair and antioxidant responses, aging, regulation of transcription, apoptosis, inflammation, and survival [5,6,7]. In this review we focus on the biological functions of Sirt1 and we also provide an insight into its role in preventing endothelial dysfunction, as well as the therapeutic strategies of targeting Sirt1 to counteract vascular aging.

## 2. Sirt1

Sirt1 was the first sirtuin to be discovered and characterized, and thus has been extensively studied. The human SIRT1 gene is located on the long arm of chromosome 10 in position 21.3 which encodes for a protein composed of 747 amino acids [7]. The structure of Sirt1 is analogous to other mammal sirtuins. The structure of Sirt1 includes a highly conserved NAD^+^-dependent sirtuins core domain and a nuclear localization signal (KRKKRK) at amino acids 41–46. Moreover, the enzyme also includes a conserved catalytic core of 275 amino acids and an N-terminal nuclear localization signal [8]. The catalytic core is composed of a Rossmann-fold domain coupled with a minor zinc finger domain, in which the zinc ion (Zn^2+^) coordinates tetrahedrally with the thiol groups belonging to four cysteines. Although the zinc ion does not participate in the catalytic activity, its presence is crucial for Sirt1 activity [1].

The main activity of Sirt1 is the deacetylation of both histones and non-histone proteins. By deacetylating these proteins, the enzyme can influence the DNA-histone interaction, thus controlling gene transcription. Indeed, Sirt1 does not bind DNA directly, but interacts with several factors associated with DNA which facilitate the correct placing of Sirt1, whose activity results in the induction of facultative or constitutive heterochromatin [9]. Sirt1 deacetylates lysines at the N-terminal tails of H3 and H4. Its deacetylation activity is mainly directed to H4K16, and at a lower rate to H3K9, H3K14, H4K8, H4K12. Moreover, it can deacetylate also the linker histone H1 at Lys26 (H1K26) [10]. Among the non-histone proteins, Sirt1 can exert its deacetylation activity also on the well-known tumor suppressor p53. Indeed, the deacetylation of p53 at K382 inhibits its nuclear translocation, thus affecting p53 transcription-dependent and independent apoptosis. Upon deacetylation, p53 shifts onto the outer membrane of mitochondria and triggers the release of the pro-apototic BCL and BAX proteins, which in turn trigger the release of cytochrome c by the mitochondria, thus starting the p53 transcription-independent apoptosis [11].

It has been reported that Sirt1 can also exert its deacetylation activity towards forkhead transcription factor O (FoxO), a key protein for modulating apoptosis, cell differentiation, cell cycle arrest, DNA repair response, and oxidative stress-resistance. The deacetylation of FoxO3 and FoxO4 is vital for aging since it results in a decrease in FOXO-induced apoptosis and in an enhanced FoxO-induced cell cycle arrest [12,13,14]. Another non-histone substrate of Sirt1 activity is peroxisome proliferator-activated receptor-γ coactivator-1α (PGC-1α), an important controller of transcription factors and also a key regulator of biogenesis of mitochondria [15]. The deacetylation of PGC-1α results in an enhanced biogenesis of mitochondria and is protective against neuronal damage and in ischemic heart disease [16].

Inflammation is an important process involved in body healing, but it also plays a key role in different diseases, including diabetes, cardiovascular disease, cancer, disease of the joints, chronic obstructive pulmonary disease, and allergies [17,18,19,20,21,22,23,24]. The nuclear factor κB (NF-κB) pathway plays a key role in regulating this process [25]. It has been reported that Sirt1 can also deacetylate NF-κB complex which inhibits the NF-κB signaling and increases oxidative metabolism resulting in suppression of inflammation. However, due to an antagonistic crosstalk, NF-κB itself can downregulate Sirt1 activity via miR-34a, IFNγ, and reactive oxygen species (ROS), promoting inflammatory responses as observed in several metabolic- or age-related disorders [26].

Sirt1 intracellular localization may change depending on the age of the cell type; for instance, in young mice, Sirt1 is expressed in the nucleus of cardiomyocytes, whereas in adult mice its expression can be detected both in the nucleus and cytoplasm [27].

Post-translational modifications (PTM) such as phosphorylation, ubiquitination, SUMOylation, and S-nitrosylation, play significant roles in modulating the expression levels, enzymatic activity, and function of Sirt1 [28,29,30]. Sirt1 can undergo phosphorylation in multiple sites at C-terminus and N-terminus triggered by the serine/threonine kinases cyclin-dependent kinase 5 (CDK5), and c-Jun N-terminal kinase (JNK) [31,32,33]. It has been reported that CDK5 can phosphorylate Sirt1 at Ser47 promoting endothelial senescence and thus, inhibition of CDK5 resulted in exerting a protective effect towards the development of cell senescence [34]. The homeodomain-interacting protein kinase 2 (HIPK2), a DNA damage response enzyme, can phosphorylate Sirt1 at Ser682 which suppresses its activity without altering the protein expression level. Moreover, Sirt1 can be phosphorylated also by the Janus kinase 1 (JAK1) at Tyr280 and Tyr301, a PTM which does not modify Sirt1 deacetylase activity but is required for Sirt1 interaction with the transcription factor STAT3 [35,36]. The Sirt1 protein level can be modulated by the activity of the E3 ubiquitin ligase SMURF2. SMURF2 ubiquinates Sirt1, triggering its degradation. Analogously, the E2-conjugating enzyme Ube2v1 triggers Ubc13-mediated Sirt1-ubiquitination and degradation, whereas the ubiquitination-mediated degradation of Sirt1 is prevented when the enzyme is bound to the ring finger protein 219 [37,38,39,40]. It has been reported that Sirt1 can be SUMOylated at Lys734, resulting in an increased catalytic activity and protein stability, whereas mutation of Sirt1 at Lys734 or desumoylation by the nuclear desumoylase Sentrin-specific protease 1 (SENP1) resulted in a reduced deacetylase activity [41]. The deacetylase activity of Sirt1 can be diminished also following S-nitrosylation, at Cys387 and Cys390, resulting in enhanced apoptosis and inflammation by increased acetylation of p53 and p65 [42,43,44]. It has been reported that following stress conditions, Sirt1 is dynamically modified by O-GlcNAcylation at Ser549 in its C-terminus, which boosts its deacetylase activity, protecting cells from stress-induced apoptosis [45]. Among the possible PTMs, the S-glutathionylation occurring at Cys67, Cys268, and Cys623 exerts a negative effect on Sirt1 activity, as well as carbonylation [46,47]. Finally, cystathionine β-synthase and cystathionine ɣ-lyase may be responsible for the indirect S-sulfhydration of 2 CXXC motifs located in the catalytic domain of SIRT1 via hydrogen sulfide generation, which results in an increased protein stability and an enhanced deacetylase activity [48,49].

## 3. Biosynthesis of NAD^+^ and Sirt1 Regulation

SIRT1 is known as a stress and energy sensor that can be activated by an increased NAD^+^/NADH ratio [50]. NAD^+^ is a vital molecule for a number of cellular reactions and functions such as cellular bioenergetics, metabolism, and survival. Indeed, it is involved in redox reactions, whereas NADH, which is the reduced form of NAD^+^, works as an electron donor, and participates in a number of reactions as a cosubstrate [51,52]. Since many enzymes utilize NAD^+^ as substrate, the levels of this molecule are crucial and influence directly and indirectly a large number of metabolic pathways. NAD^+^ levels influence the activity of the enzymes involved in cell metabolism, DNA repair, regulation of gene expression, mitochondrial activity, redox reactions, inflammation, intracellular molecules trafficking, aging, and apoptosis [53,54]. As a consequence, several pathological conditions are characterized by altered intracellular NAD^+^ levels, including cardiovascular diseases, obesity, neurodegenerative diseases, cancer, and aging [55,56,57]. In particular, age-related impairments in endothelial cells result in vascular dysfunction which promotes the rise of pathological disorders associated with old age [58].

**Figure 1:**
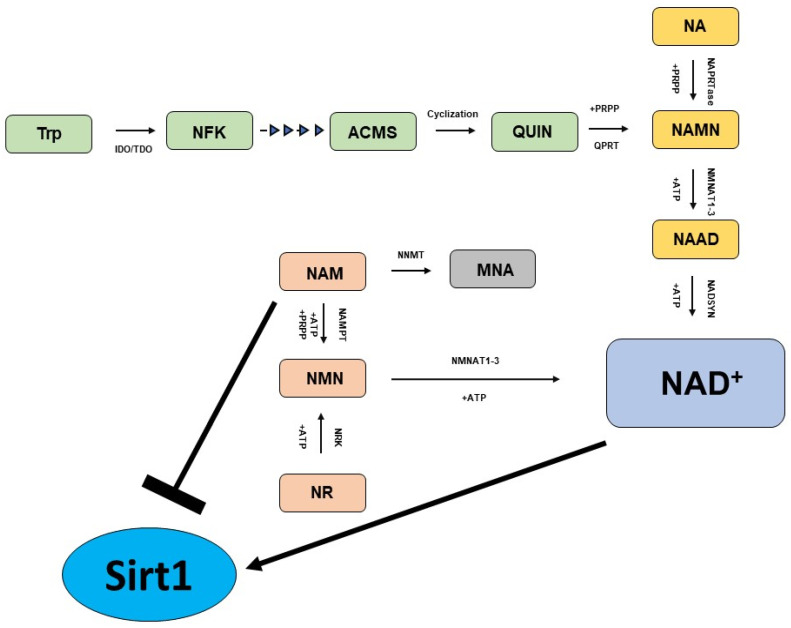
Key pathways in NAD^+^ biosynthesis. The Preiss-Handler pathway (yellow); de novo biosynthesis pathway (green); and NAD^+^ salvage pathway (light brown).

The biosynthesis of NAD^+^ can start from amino acid tryptophan (Trp), nicotinic acid (NA), nicotinamide (NAM) and nicotinamide riboside (NR), which involve distinct metabolic pathways to generate the same molecule (Figure 1) [59,60].

The Preiss–Handler pathway generates NAD^+^ utilizing NA as a starting molecule. In the Preiss–Handler pathway, the nicotinic acid phosphoribosyltransferase (NAPRTase) transforms NA to nicotinic acid mononucleotide (NAMN), utilizing 5-phosphoribosyl-1-pyrophosphate (PRPP) as a co-substrate and generating pyrophosphate (PPi) as a byproduct. In the next steps, the enzymes nicotinamide mononucleotide adenylyltransferases 1–3 (NMNATs 1–3) catalyze the conversion of NAMN into NA adenine dinucleotide (NAAD), a molecule that is finally converted into NAD^+^ by the enzyme NAD synthase (NADSYN). NAD^+^ can also be generated starting from Trp, which is first converted into N-formylkynurenine (NFK) by the enzyme indoleamine 2,3-dioxygenase (IDO) or tryptophan 2,3-dioxygenase (TDO), and upon four subsequent reaction steps it is converted into the unstable molecule α-amino-β-carboxymuconate-ε-semialdehyde (ACMS), that spontaneously undergoes cycling, generating quinolinic acid (QUIN). Subsequently, QUIN is converted into NAMN by the catalytic activity of quinolinate phosphoribosyltransferase (QPRT) using 5-phosphoribosyl-1-pyrophosphate as a cosubstrate. Finally, NAMN can be transformed into NAD^+^ via the Preiss–Handler pathway. The so-called “NAD^+^ salvage pathway” is a metabolic route that allows cells to synthesize NAD^+^ starting from NAM, which is first converted into the intermediate nicotinamide mononucleotide (NMN) by nicotinamide phosphoribosyltransferase (NAMPT), which utilizes ATP and PRPP as a cosubstrate. In the last steps, NMNAT converts NMN to NAD^+^. The last main metabolic route for the biosynthesis of NAD^+^ takes place utilizing NR, which undergoes phosphorylation catalyzed by NR kinases (NRK1/NRK2) generating NMN, a molecule that is finally converted to NAD^+^ by NMNATs in the last step.

It has been reported that specific concentrations of NAM and NAD^+^ exert a critical impact on cell survival, although excessively elevated concentrations of NAM have been proven to be detrimental [61]. For instance, it has been demonstrated that niacin administration in primary human aortic endothelial cells enhances intracellular NAD^+^ levels, which in turn activate Sirt1 activity, resulting in improved nitric oxide (NO) bioavailability [62]. There is a strict interplay between NAM and Sirt1 activity. NAM is known to be a potent inhibitor of Sirt1 activity, since it is a product reaction of Sirt1 when the enzyme transfers the acetyl residue from the acetyllysine residue of histones to the ADP-ribose component of NAD^+^ [63,64]. In this context, the enzyme nicotinamide N-methyltransferase (NNMT) plays a key role. NNMT catalyzes the methylation of NAM, yielding 1-methylnicotinamide (MNA) [65]. By methylating NAM, it reduces the intracellular concentration of NAM, which at high levels would inhibit Sirt1 activity. Indeed, the use of NAM as an inhibitor of both Sirt1 and poly-ADP-ribose polymerases has been proposed for cancer chemoprevention and therapy, especially in those malignancies where NNMT has been reported to be upregulated [66,67,68]. Accordingly, it has been reported that in endothelial cells, the NNMT inhibition resulted in nuclear Sirt1 downregulation and upregulation of the phosphorylated Sirt1 on Ser47, suggesting that the endothelial NNMT/Sirt1 pathway exerts a cytoprotective role protecting endothelial cell viability [69]. In addition, several studies demonstrated a positive effect of both NNMT and MNA, exerting vasoprotective, anti-inflammatory and anti-thrombotic activities [70,71,72]. However, some studies showed that although NAM can act initially as an inhibitor of Sirt1, it might subsequently boost Sirt1 activity due to its conversion to NAD^+^ through the NAD^+^ salvage pathway; thus, further studies are required to elucidate these aspects [73,74,75].

## 4. Endothelial Dysfunction

The endothelium is constituted by a monocellular layer that covers the inner surface of blood vessels. It crucially contributes to maintaining vascular homeostasis through different protective mechanisms, including regulating permeability and vascular tone, and exerts anti-inflammatory, antioxidant, anti-proliferative, and anti-thrombotic functions [76,77,78,79]. Indeed, the endothelium can induce the release of molecules with auto-, para- or endocrine activity, such as NO, prostacyclin, C-type natriuretic peptide (CNP), and endothelium-derived hyperpolarizing factor. By secreting these molecules, the endothelium contributes to inhibiting smooth muscle cell proliferation and migration, platelet adhesion and aggregation, and the fine regulation of biological pathways associated with thrombogenesis [80,81]. Endothelial dysfunction is characterized by a reduced synthesis and/or bioavailability of the vasodilator NO coupled with impairments due to inflammation, senescence, and oxidative stress, thus being a key factor for the development of most cardiovascular diseases (CVDs), including atherosclerosis [82]. Due to aging, several changes occur in the vasculature triggered by systemic endothelial dysfunction and amplified rigidity of large arteries [83,84]. It has been reported that endothelial dysfunction is an initial occurrence of early vascular aging that progresses in aging vessels and that can arise also in the absence of apparent CVDs or established risk factors [85,86]. Thus, the deteriorated endothelial function due to aging is important, not only from a diagnostic and pathophysiological perspective, but is also displays significant therapeutic potential.

## 5. Sirt1 and Endothelial Aging

**Figure 2:**
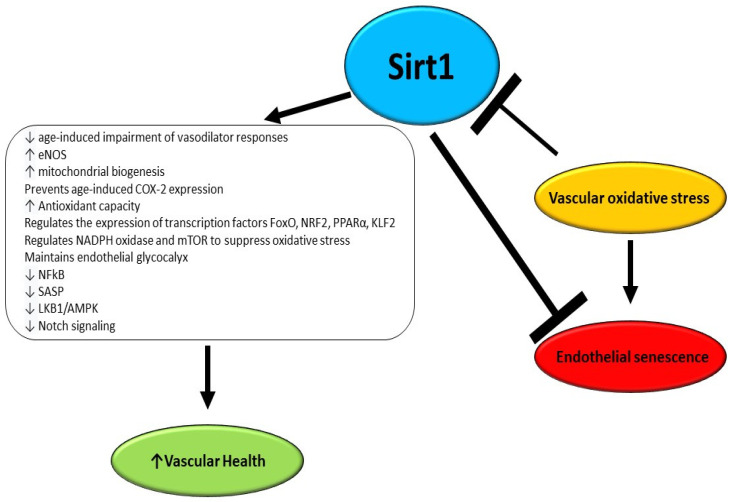
Effects of Sirt1 as anti-aging and anti-senescence factors in endothelium.

Vascular aging involves arteriosclerosis, atherosclerosis, and vascular calcifications and is accelerated by several chronic disorders, and environmental and lifestyle factors [87]. There is established evidence that an early decline in Sirt1 levels is associated with early microvascular dysfunction in adulthood with a consequently increased risk of developing CVD (Figure 2) [88]. A study performed by Guo et al. utilizing endothelial Sirt1-deficient mice demonstrated that downregulation of soluble guanylyl cyclase due to aging and upregulation of cyclooxygenase (COX)-2 in arteries is in part a consequence of the loss of endothelial Sirt1 function. Moreover, it was reported that the overexpression of the enzyme in the endothelium counteracts the impairment of vasodilator responses due to aging and suppresses vasoconstrictor reactions to acetylcholine, enhancing Notch signaling to upregulate soluble guanylyl cyclase-β1 in smooth muscle cells [89].

It has been reported that Sirt1 directly impacts the endothelial function of arteries by deacetylating endothelial NO synthase (eNOS), which in turn is activated and preserves vascular homeostasis through NO production. Consistently, the inhibition of Sirt1 in the endothelium of arteries inhibits endothelium-dependent vasodilation and decreases bioavailable NO [90]. Nonetheless, it has not been elucidated yet whether the Sirt1-mediated deacetylation of eNOS has an impact on its phosphorylation status and on the consequent NO release [91]. An elegant study performed by Bai et al. demonstrated that the overexpression of human Sirt1 in the endothelium in eNOS-deficient mice is protective against hypertension and counteracts adverse arterial remodeling occurring in aging vessels [92]. In mice undergoing calorie restriction, an increase was reported in mitochondrial biogenesis, oxygen consumption, and adenosine triphosphate production, and notably a higher expression of Sirt1 was detected. However, in eNOS-knockout mice, calorie restriction-induced expression of Sirt1 was diminished, suggesting a mutual regulatory loop involving the two enzymes in the endothelium [93].

The aging of the vasculature is a progressive event in which an impairment in the relative involvement of NO and other mediators, such as hydrogen peroxide to endothelial-dependent vasodilation and vasoconstriction, occurs. Indeed, while in healthy subjects NO is the main actor for flow-mediated dilation, patients affected by CVDs display a reduced NO-mediated dilation and an augmented hydrogen peroxide contribution [94]. It has been reported that mitochondrial dysfunction occurring in the endothelium results in a weakened/weaker endothelium-dependent vasodilatation as a consequence of diminished NO availability but enhanced generation of hydrogen peroxide [95]. Notably, Sirt1 contributes to modulating mitochondrial biogenesis by enhancing the expression of PGC-1α as well as other genes. Indeed, overexpression of Sirt1 partly reverts the impaired endothelium-dependent vasodilatation in mice characterized by flawed mitochondrial function [96]. During aging, a progressive decline has been observed in Sirt1 expression coupled with an increase of COX-2 which generates vasoconstrictor molecules to boost endothelium-dependent contractions. Interestingly, the upregulation of Sirt1 does not impact eNOS function but it counteracts age-induced COX-2 expression and increases soluble guanylyl cyclase-mediated vasodilatation [89].

ROS play a key role in the onset and progression of several diseases including cardiovascular diseases, diabetes, neurodegenerative diseases, and cancer [97,98,99,100,101,102,103]. In fact, several risk factors for developing a CVD are linked to the rise of oxidative stress, due to an increase in ROS, which promotes vascular aging and endothelial dysfunction and suppresses Sirt1 expression [104,105]. It has been demonstrated that oxidant stimuli including ROS, low-density lipoprotein cholesterol, and high glycaemia can modify the Sirt1 expression level, setting the basis for sustained endothelial dysfunction, since the diminished Sirt1 level is itself a cause of enhanced ROS generation, and contributes to vascular inflammation [31,106,107]. Indeed, Yang et al. recently demonstrated that chronic Sirt1 supplementation ameliorates endothelial function and vascular compliance by boosting eNOS activity and repressing NADPH oxidase (NOX)-related oxidative stress [108]. Consistently, it has been reported that, in animal models, calorie restriction inhibits the decline of Sirt1 levels in arteries and consequent endothelial dysfunction by counteracting oxidative stress [109,110,111,112,113]. Moreover, the strict link between Sirt1 and oxidative stress was confirmed by a study in which the Sirt1 activator SRT1720 was utilized. The activation of Sirt1 was able to revert the endothelial dysfunction by diminishing oxidative stress and inflammation, potentiating also eNOS expression [114,115,116]. Interestingly, Sirt1 activation improves angiogenesis in wounds and facilitates wound healing by promoting angiogenesis, and by protecting vascular endothelial cells from oxidative stress injury [117]. There is strong evidence that Sirt1 regulates glucose and lipid metabolism through its deacetylation activity, and exerts a positive role in ameliorating insulin resistance, which together with obesity is a major cause of endothelial oxidative stress and early vascular aging [118]. Accordingly, it has been reported that the therapeutic modulation of Sirt1 ameliorates obesity- and age-related endothelial dysfunction and protects against high-fat diet-induced metabolic abnormalities [119,120]. Zhou et al. reported that dapagliflozin improves endothelial dysfunction by restoring eNOS activity and NO bioavailability, and decreasing ROS levels via Sirt1 activation in oxidative stress-stimulated endothelial cells [121]. Moreover, it has been reported that Sirt1 can exert a protective effect against oxidative stress by influencing the expression of several nuclear transcription factors such as NF-κB, FoxO, nuclear factor-erythroid-2-related factor 2 (NRF2), peroxisome proliferator-activated receptor alpha (PPAR-α), and Krüppel-like factor 2 (KLF2) [122,123,124,125]. It has been demonstrated that Sirt1 can induce the upregulation of manganese superoxide dismutase thus reducing oxidative stress [126,127]. The ability of Sirt1 to counteract oxidative stress arises also from its capacity of interaction with hydrogen sulfide, due to the inhibition of p66Shc adaptor protein expression levels, through the regulation of NOX, eNOS, and the mechanistic target of rapamycin (mTOR) [126,128,129,130,131]. Finally, Sirt1 contributes to the homeostasis of glycocalyx, which is crucial for flow-induced NO release and inhibits oxidative stress [132,133]. Despite the recent advances achieved in understanding the intricate interactions by which Sirt1 contributes to preventing vascular aging, many mechanisms related to Sirt1 protection against endothelial dysfunction are still far from being fully elucidated, thus requiring deeper insights.

## 6. Sirt1 and Endothelial Senescence

Endothelial senescence is a pathophysiological process in which the endothelium undergoes structural and functional changes and is crucial for vascular dysfunction, which leads to age-related disease [134,135,136]. Senescent endothelial cells are characterized by changes in cell size and morphology, enhanced lysosomal activity, resistance to apoptosis, decreased NO bioavailability, cell cycle arrest, and reduced proliferation. Moreover, they display augmented senescence-associated-β-galactosidase (SA-β-gal) activity, senescence-associated secretory phenotype (SASP), and increased expression of senescence-associated proteins such as P16 and Sirt1 [134,137]. While aging is characterized by a gradual functional decline, senescence is a cellular response featuring irreversible growth arrest and other phenotypic alterations that include a pro-inflammatory secretome [138,139].

Although endothelial senescence and endothelial dysfunction are mainly initiated by oxidative stress and inflammation, the exact molecular regulatory mechanisms underlying this process remain largely undisclosed [95]. It has been reported that endothelial cell senescence occurs following a number of replication cycles or as a consequence of excessive stress stimulation. Indeed, an accumulation of senescent endothelial cells in arteries during aging has been demonstrated, and in pathological conditions including atherosclerotic plaques and abdominal aortic aneurysm [137,140,141,142,143]. Following senescence, endothelial senescent cells contribute to sustaining a pro-inflammatory and pro-oxidative status and gradually express a SASP that boosts the establishment of a senescent microenvironment that promotes vascular aging [144,145,146,147]. It has been reported that Sirt1 is able to repress the transcriptional activity of NFkB, the main transcription factor for SASP, through the interaction with NFkB and the consequent deacetylation of RelA/p65 at lysine [95,148,149]. Hayakawa et al. reported that Sirt1 can inhibit the expression of SASP factors such as IL-6 and IL-8 through the deacetylation of histones in their promoter regions [150]. Cellular senescence is causally involved in inducing age-related phenotype and thus it should not surprise that removing senescent cells counteracts or delays tissue dysfunction and prolongs lifespan [151]. Endothelial senescence promotes the establishment of endothelial dysfunction, a pro-inflammatory, pro-oxidant and pro-thrombotic condition, and reduces the regeneration capacity of the endothelium, thus playing a crucial role in promoting atherogenesis and age-related vascular disorders [136,152,153,154]. Indeed, human artery endothelial cells that have become senescent arrest the cell cycle preventing further divisions and acquire typical phenotypic traits causing impairment of the angiogenic process, vascular inflammation, and remodeling, and display increased levels of ROS and a decreased NO bioavailability [144,155,156]. In this regard, the accumulation of endothelial senescent cells in the vasculature is a serious clinical problem and several senolytic molecules have been designed in order to mitigate the SASP effects [157,158,159].

It has been reported that Sirt1 displays anti-senescence properties in a wide range of mammalian cells, and that Sirt1 inhibition triggers premature senescence-like replication arrest in human cancer cell lines, characterized by senescence-associated beta-galactosidase activity and increased expression of plasminogen activator inhibitor 1. Moreover, the senescence status was accompanied by an altered activation of some mitogen-activated protein kinase (MAPK) pathways, such as extracellular-regulated protein kinase, c-jun N-terminal kinase and p38 MAPK, in response to epidermal growth factor (EGF) and insulin-like growth factor-I (IGF-I) [160]. Moreover, a study performed by Zheng et al. demonstrated that Sirt1 inhibition by nicotinamide induced a premature senescence of human umbilical vascular endothelial cells (HUVECs), whereas a Sirt1 overexpression triggered by sodium hydrosulfide was able to delay senescence of HUVECs induced by NAM [161]. A recent study performed by Tai et al. showed that the administration of dapagliflozin protects endothelial cells against the rise of senescence by regulating Sirt1 signaling in diabetic mice [162]. In primary porcine aortic endothelial cells (PAECs), which underwent senescence after several passages, an increased phosphorylation of Sirt1 at serine 47 was detected, a post-translational modification that exerts an inhibitory effect on biological functions of Sirt1 in endothelial cells [31]. In line with these findings, the endothelial cells of transgenic mice with endothelial-specific overexpression of Sirt1 were found to be more resistant to paraquat-induced vascular senescence [163]. Senescent PAECs showed enhanced levels of both liver kinase B1 (LKB1), a serine/threonine kinase that also acts as a tumor suppressor, and phosphorylated AMP-activated protein kinase (AMPK), a downstream target of LKB1. This evidence suggests that Sirt1 represses LKB1/AMPK signaling resulting in the promotion of survival and proliferation, and thus inhibiting senescence in endothelial cells [163]. In the nucleus, Sirt1 binds to the DOC domain of HECT and RLD domain containing E3 ubiquitin protein ligase 2 (HERC2) through its amino-terminus, which in turn is responsible for the ubiquitination LKB1 in the nucleus of endothelial cells. Through this mechanism, Sirt1 finely regulates the crosstalk between endothelial and vascular smooth muscle cells counteracting harmful arterial remodeling and maintaining vascular homeostasis [92].

It has been reported that Sirt1 deficiency in endothelial cells promotes the development of fibrosis and induces aberrant secretome, mainly related to activated Notch signaling, which is known to promote senescence, increased permeability and pro-inflammatory responses [89,164,165,166,167]. Indeed, it has been demonstrated that Notch pathway components are upregulated in luminal endothelial cells of atherosclerotic lesions in both mouse and human aortas, as well as in aged but not in young endothelial cells. The upregulation of Notch pathways results in significantly upregulated expression of several molecules implicated in the inflammatory response such as IL-6, IL-8, IL-1α, RANTES and ICAM-1 [168]. In this context, it is noteworthy that Sirt1-deficient endothelial cells are characterized by a marked activation of Notch signaling, proven by the overexpression of delta-like ligand 4 (DLL4), Notch intracellular Domain (NICD) and Notch target genes such as Hey1 and Hes1 [167]. However, although it has been reported that an overexpression of Sirt1 in smooth muscle cells upregulates the Notch signaling to upregulate soluble guanylyl cyclase, which contrasts COX-2, thus preventing vascular aging, it is still unclear whether Sirt1 counteracts the rise of senescence by inhibiting Notch signaling [89].

It is well known that Sirt1 plays a key role in maintaining genome stability which is one of the major mechanisms by which accumulating DNA damage during cell replications leads to accumulation of senescent endothelial cells in arteries [169,170,171]. Indeed, upon DNA damage, the activation of Sirt1 exerts a protective effect against p53-induced senescence or apoptosis by interacting with poly-ADP ribose and forms molecular complexes with key factors such as checkpoint kinase 2 (CHEK2), BRCA1/BRCA2-associated helicase 1, tumor suppressor p53-binding protein 1, Werner helicase, BRG1, and H2AX [172,173,174,175].

Finally, another mechanism by which endothelial cells undergo senescence is the telomere shortening or capping [140,176,177]. It has been reported that Sirt1 is able to interact with telomeric repeat-binding factor 2-interacting protein 1 (TERF2IP), a regulator of both telomere function and NFkB signaling, preventing the nuclear-cytoplasmic shuttle of TERF2IP which results in a suppressed activation of NFkB and an upregulation of COX-2 [31,178]. It has been demonstrated that cyclin-dependent kinase 5 hyperphosphorylation of Sirt1 at the serine 47 residue blocks the antisenescence activity of Sirt1, and plays a pivotal role in the loss-of-Sirt1 function occurring in vascular aging. Indeed, the use of roscovitine, a cyclin-dependent kinase 5 inhibitor, prevents the development of cellular senescence and atherosclerosis in mice [33]. The anti-aging effects of Sirt1 have also been linked to the aging-suppressor protein Klotho. Indeed, it was demonstrated that Klotho deficiency downregulates Sirt1 activity in endothelial cells [179]. Taken together, these pieces of evidence confirm that vascular aging occurs in part also due to endothelial senescence and thus, current efforts are directed to the development of senotherapeutics which can be senolytics, drugs that can remove senescent cells, or senomorphics, which are molecules able to repress their SASP secretome [180,181,182,183,184]. Given the above-mentioned roles of Sirt1, this enzyme is an excellent candidate for preventing cellular senescence, in order to develop Sirt1-based therapies to reverse endothelial cell senescence and vascular aging.

## 7. Conclusions

Due to its crucial role in vascular aging, Sirt1 represents a promising therapeutic target. Indeed, through its antioxidant and anti-inflammatory effects, and by improving endothelium-dependent vasodilatation, Sirt1 prevents arterial aging. Discovering natural compounds or designing and developing synthetic drugs that could be effective and safe molecules for achieving Sirt1 activation could be an essential strategy for preventing and treating vascular aging. In this context, identifying nutraceutical regimens providing Sirt1-activating agents may also have significant importance for health promotion.

## Data Availability

No new data were created or analyzed in this study. Data sharing is not applicable to this article.
